# mmquant: how to count multi-mapping reads?

**DOI:** 10.1186/s12859-017-1816-4

**Published:** 2017-09-15

**Authors:** Matthias Zytnicki

**Affiliations:** MIAT, Toulouse INRA, BP 52627, Castanet-Tolosan cedex, 31326 France

**Keywords:** RNA-Seq, Quantification, Multi-mapping reads

## Abstract

**Background:**

RNA-Seq is currently used routinely, and it provides accurate information on gene transcription. However, the method cannot accurately estimate duplicated genes expression. Several strategies have been previously used (drop duplicated genes, distribute uniformly the reads, or estimate expression), but all of them provide biased results.

**Results:**

We provide here a tool, called mmquant, for computing gene expression, included duplicated genes. If a read maps at different positions, the tool detects that the corresponding genes are duplicated; it merges the genes and creates a merged gene. The counts of ambiguous reads is then based on the input genes and the merged genes.

**Conclusion:**

mmquant is a drop-in replacement of the widely used tools htseq-count and featureCounts that handles multi-mapping reads in an unabiased way.

**Electronic supplementary material:**

The online version of this article (doi:10.1186/s12859-017-1816-4) contains supplementary material, which is available to authorized users.

## Background

**Fig. 1:**
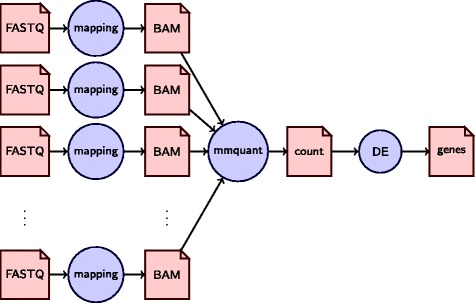
Possible bioinformatics pipe-line for RNA-Seq differential expression analysis. The analysis starts with several FASTQ files, produced by the sequencing of several replicates of two conditions (e.g. wild type *vs* mutant). When a genome is available, the reads are mapped, e.g. with STAR [15], and the corresponding positions are stored into a BAM file. A quantification tool, such as mmquant, presented here, counts the number of reads per gene. Statistical test for differential expression is performed by a third tool, like DESeq2 [6]

RNA-Seq has emerged as the standard method to analyze several genes in one experiment. Among the different contexts in which RNA-Seq is used, differential gene expression is arguably the most common. This method can be decomposed into several steps, although variations exist: read mapping, gene quantification, and test for differential gene expression (see Fig. 1). Gene quantification aims at estimating the level of expression of a gene, given the number of reads that map to this gene.

In complex genomes, many genes are duplicated, and they constitute the majority of the genes in polyploid genomes such as wheat. In this configuration, a read produced by a duplicated gene may be mapped equally well to each homologous gene, giving rise to multi-mapping reads. These reads are complex to use in the quantification step, and several methods have been used to circumvent this problem.

**Fig. 2:**
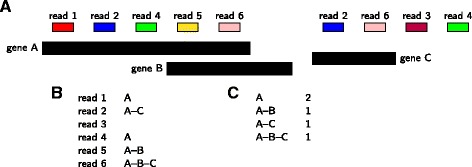
Overview of the method, on an example. **a**: A toy configuration, with three genes in black: A, B, and C. Notice that A and B overlap. Six reads have been mapped to the genome, some of them (reads 2, 4, and 6) map at two different locations. If a read maps unambiguously to a unique locus and matches a unique gene (like read 1), we attribute the corresponding gene to the read (here, gene A). If a read, like read 2, matches two different genes, we create a “merged” gene, here A–C, and attribute this merged gene to the read. If a read (read 3) does not match any gene, it is not used. If a read (read 4) matches a gene (gene A) and an intergenic region, the read is attributed to the gene only. If a read (read 5) matches two different genes because the genes overlap, the read is also attributed to the merged gene (gene A–B). Similarly, if a read (read 6) matches two overlapping genes and an other gene, the three genes are merged (A–B–C). Table **b** provides the attributed gene for each read. **c** is the quantification table and the output of the tool for this configuration. Genes are sorted in lexicographical order, as shown in the example. As a consequence, merged gene (A, B) will always be displayed as A–B, and never as B–A

For instance, the authors of featureCounts [1] designed in-built annotations, that have merged duplicated and overlapping genes. Recently, [2] proposed an on-line method, that requires no pre-processing of the annotation. If a read maps to gene A and gene B, a new “merged gene” is created, and the read is attributed to this merged gene, named gene A–B (see Fig. 2). The method provides counts for the genes given in input and for the merged genes. Merged genes can be used as standard genes in the downstream analysis. This method uses all the information given by the RNA-Seq sequencing on ambiguous reads, without any assumption nor inference.

The designers of the method [2] provided a prototype that implemented the approach. However, the prototype supposes that the read and the annotation files have a pre-defined format. Fine-tuning parameters, available in most standard quantification tools, are missing (e.g. overlap type and library type, see *infra*). Last, it involves several Perl files and thus is slow, not appropriate for large datasets involving many RNA-Seq experiments. In this paper, we present a new tool that implements the aforementioned method, together with state-of-the-art characteristics in terms of usability, especially speed and options. We believe that this tool will be useful to the RNA-Seq users, because it is simple (a drop-in replacement of the highly used featureCounts [1] or htseq-count [3]) and more informative, with no drawback.

The rest of the paper is organized as follows. We will first detail the implementation of our tool. Then, we will compare the results given by our tool and other state-of-the-art tools. We will conclude by mentioning future directions.

## Implementation

Briefly, the default method supposes that the reads have been sorted beforehand. Many other tools, such as visualization tools, have the same requirement, and, arguably, this step should be performed anyway. Since both input sets are sorted, the search is approximately linear with respect to the size of the input and the size of the output. If the reads are not sorted, we sort the genes into a vector, cut the genome into non-overlapping bins (default size: approximately 10kb) and store the index of the first gene in or after each bin. For each read, we scan the genes starting from the position indexed by the bin of the 5’-most position of the read.

For comparison, htseq-count requires that the reads be sorted either by position or by name. featureCounts requires paired-end reads to be sorted by name.

mmquant proceeds in two steps for quantification. The first step decides whether a read *matches* a gene it overlaps with. Depending on the value *ℓ* of the “-l” parameter, provided by the user, the read matches the gene iff:

- *ℓ*≤0 and the read is totally included in the gene;
- *ℓ*≥1 and *n*≥*ℓ*, where *n* is the number of overlapping base pairs between the read and the gene;
- 0<*ℓ*<1 and *n*≥*ℓ*×*s*, where *n* has been previously defined, and *s* is the size of the read.

The first step is the search of all the matching genes for a given read. If a read maps at several locations, the mapping tool sets the NH tag (that provides the number of hits) of the SAM/BAM files to a value greater than one. mmquant uses this information and keeps the read and the matching genes in memory until all the hits have been scanned.

**Fig. 3:**
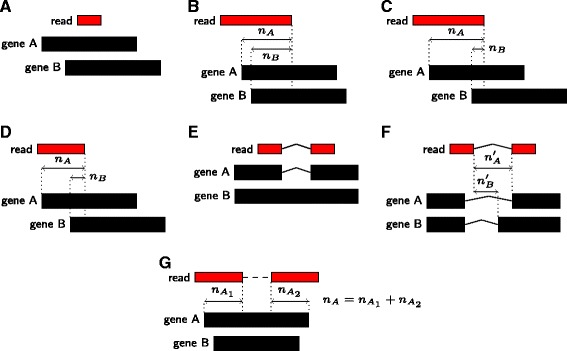
Ambiguous reads resolution. **a**: The read is included in both genes A and B. Here, the resolution cannot be solved, and the read will be attributed to gene A–B. **b**: The read is not totally included in gene A, neither in gene B. *n*_*A*_ nucleotides of the read overlap with gene A, and *n*_*B*_ overlap with gene B, and *n*_*A*_>*n*_*B*_. If *n*_*A*_≫*n*_*B*_, we may attribute the read to gene A only. However, if *n*_*A*_≈*n*_*B*_, the ambiguity cannot be resolved, and the read is attributed to A–B. The two following cases show the rules to resolve ambiguity. **c**: We suppose here that *n*_*A*_>*n*_*B*_+*N*, where *N* is a parameter set by the user (default: 30). In this case, mmquant will attribute the read to gene A only. **d**: We suppose here that *n*_*A*_>*n*_*B*_×*P*, where *P* is given by the user (default: 2). The read will be attributed uniquely to gene A. **e**: Here, the single end read contains an intron. Exon-wise, the read can be attributed to gene A or B. In case of ambiguity, introns are compared. The intron of the read matches the intron of gene A, whereas gene B has no intron there. The read is thus attributed to A. **f**: The read is ambiguous exon-wise. We compute *nA*′ and *nB*′, the number of nucleotides shared by the intron of the read and the introns of genes A and B respectively. Ambiguity is solved using *nA*′, *nB*′ and the rules given in **c** and **d**. **g**: The read is paired-end. In case of ambiguity, *n*_*A*_ and *n*_*B*_ are computed as the sums of the overlapping bases between the two reads and the gene A and B respectively. The rules presented in **c** and **d** apply next

The second step resolves ambiguities. When a read matches several genes, some matching genes can be possibly discarded, depending on the number of overlapping base pairs. The rules that mmquant uses are provided in Fig. 3.

The tool supports paired-end reads, and checks that both ends may match the same transcript, in a way that is consistent with the sequencing strategy (forward–reverse, reverse–forward, etc.). The fragments (i.e. the pairs of reads) are then counted for quantification.

For comparison, htseq-count has three modes: “union”, “intersection-strict” and “intersection-nonempty”. The reader can consult the htseq-count article [3] for a description of these modes. mmquant can emulate the “union” —the recommended mode— and the “intersection-strict” modes, with parameters “-l 1” and “-l -1” respectively, but not the “intersection-nonempty”. Ambiguous reads are not used for quantification. In featureCounts, two parameters (“minOverlap” and “fracOverlap”) are used instead of our “-l” parameter. Default strategy discards multi-matching reads. These reads can be used for quantification when the options “-M” and “-O” are set. In this case, a read will be attributed to each matching gene (with a normalized weight if the option “fraction” is used). However, this practice is discouraged because it almost always provides biased results. Besides, the “largestOverlap” option makes it possible to assign a read to the gene that has the largest number of overlapping bases. This strategy can be emulated by mmquant.

## Results

We tested our method on recent data, taken from [4]. Briefly, this article uses RNA-Seq of human brain to find genes that are differentially expressed in individuals diagnosed with bipolar disorder. The article uses two sequencing protocols (GA-IIx and HiSeq) and microarray to validate the results. Our aim is not to reproduce their pipe-line (parameters and software versions have not been provided in the article), but to show the differences between the most used gene quantification tools (htseq-count and featureCounts), and our tool. We focused on the HiSeq dataset, that contained 6 replicates in each condition, because HiSeq is probably the most used sequencing machine now. Reads are 100 bp (base pairs) long, each sample is paired-end and contains approximately 200 millions reads. Admittedly, this dataset is challenging because duplicated genes are known to play a major role in human brain [5].

Our pipe-line uses STAR v2.5.0a (with parameters recommended by ENCODE) and DESeq2 v1.14.1 [6] with default options and adjusted *p*-value at 5%, whereas [4] used TopHat, htseq-count and DESeq (arguably the reference tools at the time of publication). In the original paper, the author found only 11 differentially expressed genes (with adjusted *p*-value at 5%). We found all of them with our pipe-line and all the quantification tools, except one, a pseudo-gene (probably due to a problem of TopHat, which, on the earliest versions, was known to favor pseudo-genes with no intron compared to mature transcripts).

htseq-count was used in the “union” mode because it is the recommended one. As a consequence, if a read overlaps two genes, htseq-count considers that the read is ambiguous and does not use it for quantification. featureCounts was used with options “-p -B -C” and mmquant was used with the “-l 1” parameter (which compares a gene and a read as soon as they overlap by 1 bp).

Strikingly, the *p*-values obtained with the three different quantification strategies show a great variability (see Additional file 1). htseq-count, featureCounts and mmquant (excluding merged genes) gave 734, 835 and 763 differentially expressed genes respectively. Most of the difference comes from the way reads are assigned to the genes. htseq-count and mmquant may attribute a read to a gene as soon as they have at least one common base pair, whereas featureCounts requires the read to included in the gene. Consequently, htseq-count and mmquant count more reads per gene than featureCounts. Moreover, mmquant has several rules to resolve ambiguities, such as reads matching several genes, whereas htseq-count and featureCounts discard such reads. As a consequence, mmquant finds more reads per gene than htseq-count. Then, mmquant tests more genes (non-merged and merged genes) than the other tools. Thus, the adjusted *p*-value is higher even with the same raw *p*-value. This is a usual trade-off between specificity and sensitivity. Last, the ajusted *p*-value threshold emphasize these differences. For instance, 133 of the 158 genes that are found by featureCounts and not by mmquant have an adjusted *p*-value less that 10%, when using mmquant.

mmquant found that 5–6% of the reads where multi-mapped and could be attributed to several genes. As a consequence, it found 254 additional differentially expressed merged genes, involving 516 new genes. Note that one fourth of the differentially expressed genes is merged. We tried to find an over-represented function for the genes involved in the merged set, using DAVID [7]. However, almost half of them have no known function, probably because they are duplicated, and no over-represented function was clearly linked to bipolar disorder.

We then considered the 33 merged genes with adjusted *p*-value < 1%, which represented very good candidates. These merged genes included 75 genes that were not detected otherwise (neither by htseq-count nor featureCounts, nor in the non-merged genes found by mmquant). This gene list includes new excellent candidates with putative links to bipolar disorder, including *ADK* [8], *GTF2I* [9], *hnRNP-A1* [10], *HTRA2* [11], *PKD1* [12] and *RERE* [13], which have been linked to various brain-related diseases (see Additional file 2). Some of these genes have complex regulation systems in *cis*: *ADK* and *HTRA2* contain overlapping processed pseudogenes and antisense transcripts or genes, and mmquant merges these annotations on the fly. Other genes, like *GTF2I*, *hnRNP-A1*, *PKD1*, and *RERE*, are duplicated genes, or have produced a pseudo-gene in another locus. It is out of the scope of this study to validate these genes, but we would like to emphasize that, because these genes are duplicated, or overlap with other genes, they have been removed from the standard analysis.

Concerning time, featureCounts is the fastest tool, taking 8–11min per sample; mmquant is second with 21–29 min (+1–3 min if the reads are not sorted); htseq-count, written in Python, takes 4h15min–5h29min. mmquant is slower than featureCounts because it has to store (and look up) all the reads that have been mapped several times. We obtained this results allocating one thread per BAM file, but featureCounts can be further accelerated by allocating more than one thread per input file, whereas mmquant and htseq-count cannot.

To confirm these results, we used several differential RNA-Seq datasets available from the Gene Expression Omnibus (GEO) [14], summarized in Table 1. We used several model multicellular eukaryotes, with different sequencing types and depths. Figures here globally confirm previous results. The number of differentially expressed genes given by each tool is comparable. featureCounts and htseq-count provide near-identical results for single-end data. featureCounts is the fastest tool, mmquant is somewhat slower, and htseq-count is an order of magnitude slower. In mouse, we found that the raw counts and the (unadjusted) *p*-values of the non-merged genes were almost identical in the three methods. However, since mmquant adds a large number of merged genes, the adjusted *p*-values increase, and thus 3 differentially genes are not found by mmquant. In yeast, we observed that the added merged genes altered the count distribution: the library sizes and over-dispersion parameters are sensibly different with and without merged genes. As a consequence, mmquant finds less differentially expressed genes. We believe that our method is more accurate, because it uses more data to infer the count distributions. Notice that mmquant always finds new merged genes.

**Table 1:** Results on other datasets

Organism	*D. melanogaster*	*M. musculus*	*A. thaliana*	*S. cerevisiae*
GEO accession	GSE80323	GSE86865	GSE89850	GSE83827
reference	[16]	[17]	[18]	[19]
# exp. genes	16383	24516	25170	6722
# merged genes	7237	25302	5903	4782
type	paired	single	paired	single
# replicates	4	3	3	2
# reads	27M–33M	39M–47M	12M–16M	10M–14M
# hits	28M–34M	53M–70M	12M–15M	13M–17M
# fc genes	1446	13	4622	546
# htsc genes	1432	13	4557	546
# mm genes	1441	10	4599	388
# fc and htsc	1420	13	4503	546
# fc and mm	1415	10	4534	387
# htsc and mm	1399	10	4458	387
# mm merged only	191	2	394	97
fc time	1mn28–	1mn25–	0mn46–	0mn16–
	1mn56	2mn38	1mn10	0mn29
htsc time	36mn–	21mn–	20mn–	4mn11–
	46mn	25mn	26mn	5mn58
mm time	2mn35–	2mn06–	1mn19–	0mn20–
	3mn31	2mn42	1mn43	0mn28
mm time unsorted	3mn31–	2mn22–	1mn28–	0mn24–
	4mn18	2mn53	2mn11	0mn31

The description of each line follows. # exp. genes: number of expressed genes (at least one read in one of the replicates); # merged genes: number of merged genes found by mmquant; type: single-end or paired-end; # replicates: number of replicates in each of the two conditions; # reads: number of reads sequenced; # hits: number of hits given by STAR; # fc genes: number of differentially expressed genes found by featureCounts; # htsc genes: number of differentially expressed genes found by htseq-count; # mm genes: number of differentially expressed non-merged genes found my mmquant; # mm merged only: number of genes that constitute the differentially expressed merged genes, and that are not found by the other two methods; # fc and htsc: number of differentially expressed genes found by both featureCounts and htseq-count; fc time: time spent by featureCounts; mm time unsorted: time spent by mmquant, using unsorted data

## Conclusion

Gene quantification is an essential step of many RNA-Seq analyses. Yet, the assumption used by the quantification tools is not always fully understood, especially concerning multi-mapping reads. With mmquant, we provide a simple tool, that includes these reads in the quantification step, with no assumption on the read distribution. On our test sample, we found that these multi-mapping reads could provide up to 25% new differentially expressed merged genes. These genes were outside of the scope of previous analyses, thus biasing their results. We hope that, with mmquant, a drop-in replacement of previous tools, the genomic “dark matter” will be at last explored.

In the future, we would like to extend the concept to feature quantification. In some protocols, especially sRNA-Seq, one may want to count the number of reads per feature, such as miRNA, tRNA, rRNA, etc. Even though a read maps at several loci, all the loci may belong to the same feature. This method could help reducing ambiguities and providing useful results as well.

## Availability and requirements

- Project name: mmquant
- Project home page: https://bitbucket.org/mzytnicki/multi-mapping-counter and https://toolshed.g2.bx.psu.edu/repository?repository_id=93e0efd7b8426c9c for in the Galaxy Tool Shed
- Operating system(s): Linux
- Programming language: C++
- Other requirements: C++11 or higher, and zlib
- License: Lesser General Public License 3.0

## Additional files

Additional file 1 Comparison of the *p*-value distributions. Each square outside of the diagonal compares a couple of tools. For instance, the top-right square compares featureCounts with mmquant. Each dot is a gene, its *x*-axis value is −log of the *p*-value given by a tool, whereas the *y*-axis value is −log of the *p*-value given by the other tool. In the aforementioned square, the value on the *x*-axis is given using the mmquant strategy, and the value on the *y*-axis is given using the featureCounts strategy. All axes are log-scaled, and *p*-values have been increased by 10^5^ to render 0s. (PDF 1771.52 kb)

Additional file 2 List of differentially expressed merged genes, related to brain diseases. For each of the six “merged genes” potentially linked to brain diseases, we provide the actual genes they are made of, as well as their genomic loci. Notice that merged genes 3 to 6 involve two to three different loci. (PDF 48 kb)

## Abbreviations

BAM: Binary alignment map
bp: Base pairs
GC%: Guanine–cytosine content
GEO: Gene expression omnibus
h: Hours
kb: Kilo-base
M: Millions
mn: Minutes
qRT-PCR: Quantitative reverse transcription polymerase chain reaction
SAM: Sequence alignment map

## References

[1] Liao Y, Smyth GK, Shi W (2014). featureCounts: an efficient general purpose program for assigning sequence reads to genomic features. Bionformatics.

[2] Robert C, Watson M (2015). Errors in RNA-Seq quantification affect genes of relevance to human disease. Genome Biol.

[3] Anders S, Pyl PT, Huber W (2015). HTSeq–a Python framework to work with high-throughput sequencing data. Bionformatics.

[4] Akula N, Barb J, Jiang X, Wendland JR, Choi KH, Sen SK, Hou L, Chen DTW, Laje G, Johnson K, Lipska BK, Kleinman JE, Corrada-Bravo H, Detera-Wadleigh S, Munson PJ, McMahon FJ (2014). RNA-sequencing of the brain transcriptome implicates dysregulation of neuroplasticity, circadian rhythms and GTPase binding in bipolar disorder. Mol Psychiatry.

[5] Geschwind DH, Konopka G (2012). Neuroscience: Genes and human brain evolution. Nature.

[6] Love MI, Huber W, Anders S (2014). Moderated estimation of fold change and dispersion for RNA-seq data with DESeq2. Genome Biol.

[7] Huang DW, Sherman BT, Lempicki RA (2009). Systematic and integrative analysis of large gene lists using DAVID bioinformatics resources. Nat Protoc.

[8] Boison D (2006). Adenosine kinase, epilepsy and stroke: mechanisms and therapies. Trends Pharmacol Sci.

[9] Sakurai T, Dorr NP, Takahashi N, McInnes LA, Elder GA, Buxbaum JD (2011). Haploinsufficiency of *Gtf2i*, a gene deleted in Williams Syndrome, leads to increases in social interactions. Autism Res.

[10] Liu XY, Li HL, Su JB, Ding FH, Zhao JJ, Chai F, Li YX, Cui SC, Sun FY, Wu ZY, Xu P, Chen XH (2015). Regulation of RAGE splicing by hnRNP A1 and Tra2 *β*-1 and its potential role in AD pathogenesis. J Neurochem.

[11] Patterson VL, Zullo AJ, Koenig C, Stoessel S, Jo H, Liu X, Han J, Choi M, DeWan AT, Thomas JL, Kuan CY, Hoh J (2014). Neural-Specific Deletion of *Htra2* Causes Cerebellar Neurodegeneration and Defective Processing of Mitochondrial OPA1. PLOS ONE.

[12] Wodarczyk C, Rowe I, Chiaravalli M, Pema M, Qian F, Boletta A (2009). A Novel Mouse Model Reveals that Polycystin-1 Deficiency in Ependyma and Choroid Plexus Results in Dysfunctional Cilia and Hydrocephalus. PLOS ONE.

[13] Fregeau B, Kim B, Hernández-García A, Jordan V, Cho M, Schnur R, Monaghan K, Juusola J, Rosenfeld J, Bhoj E, Zackai E, Sacharow S, Barañano K, Bosch DM, de Vries BA, Lindstrom K, Schroeder A, James P, Kulch P, Lalani S, van Haelst M, van Gassen KI, van Binsbergen E, Barkovich AJ, Scott D, Sherr E (2017). De Novo Mutations of *RERE* Cause a Genetic Syndrome with Features that Overlap Those Associated with Proximal 1p36 Deletions. Trends Pharmacol Sci.

[14] Barrett T, Wilhite S, Ledoux P, Evangelista C, Kim I, Tomashevsky M, Marshall K, Phillippy K, Sherman P, Holko M, Yefanov A, Lee H, Zhang N, Robertson C, Serova N, Davis S, Soboleva A (2013). NCBI GEO: archive for functional genomics data sets–update. Nucleic Acids Res.

[15] Dobin A, Davis CA, Schlesinger F, Drenkow J, Zaleski C, Jha S, Batut P, Chaisson M, Gingeras TR (2013). STAR: ultrafast universal RNA-seq aligner. Bionformatics.

[16] Hateleya S, Hosamanib R, Bhardwajb SR, Pachtera L, Bhattacharyab S (2016). Transcriptomic response of Drosophila melanogaster pupae developed in hypergravity. Genomics.

[17] Liu X, Zhang Y, Ni M, Cao H, Signer RAJ, Li D, Li M, Gu Z, Hu Z, Dickerson KE, Weinberg SE, Chandel NS, DeBerardinis RJ, Zhou F, Shao Z, Xu J (2017). Regulation of mitochondrial biogenesis in erythropoiesis by mTORC1-mediated protein translation. Nat Cell Biol.

[18] Tang Y, Liu X, Liu X, Li Y, Wu K (2017). *Arabidopsis* NF-YCs Mediate the Light-Controlled Hypocotyl Elongation via Modulating Histone Acetylation. Mol Plant.

[19] Losh JS, King AK, Bakelar J, Taylor L, Loomis J, Rosenzweig JA, Johnson SJ, van Hoof A (2015). Interaction between the RNA-dependent ATPase and poly(A) polymerase subunits of the TRAMP complex is mediated by short peptides and important for snoRNA processing. Nucleic Acids Res.

