# Supplementary figures and images for: mmquant: how to count multi-mapping reads?
Source: BMC Bioinformatics. 2017 Sep 15;18:411. doi: 10.1186/s12859-017-1816-4 (PMC5603007; doi:10.1186/s12859-017-1816-4)

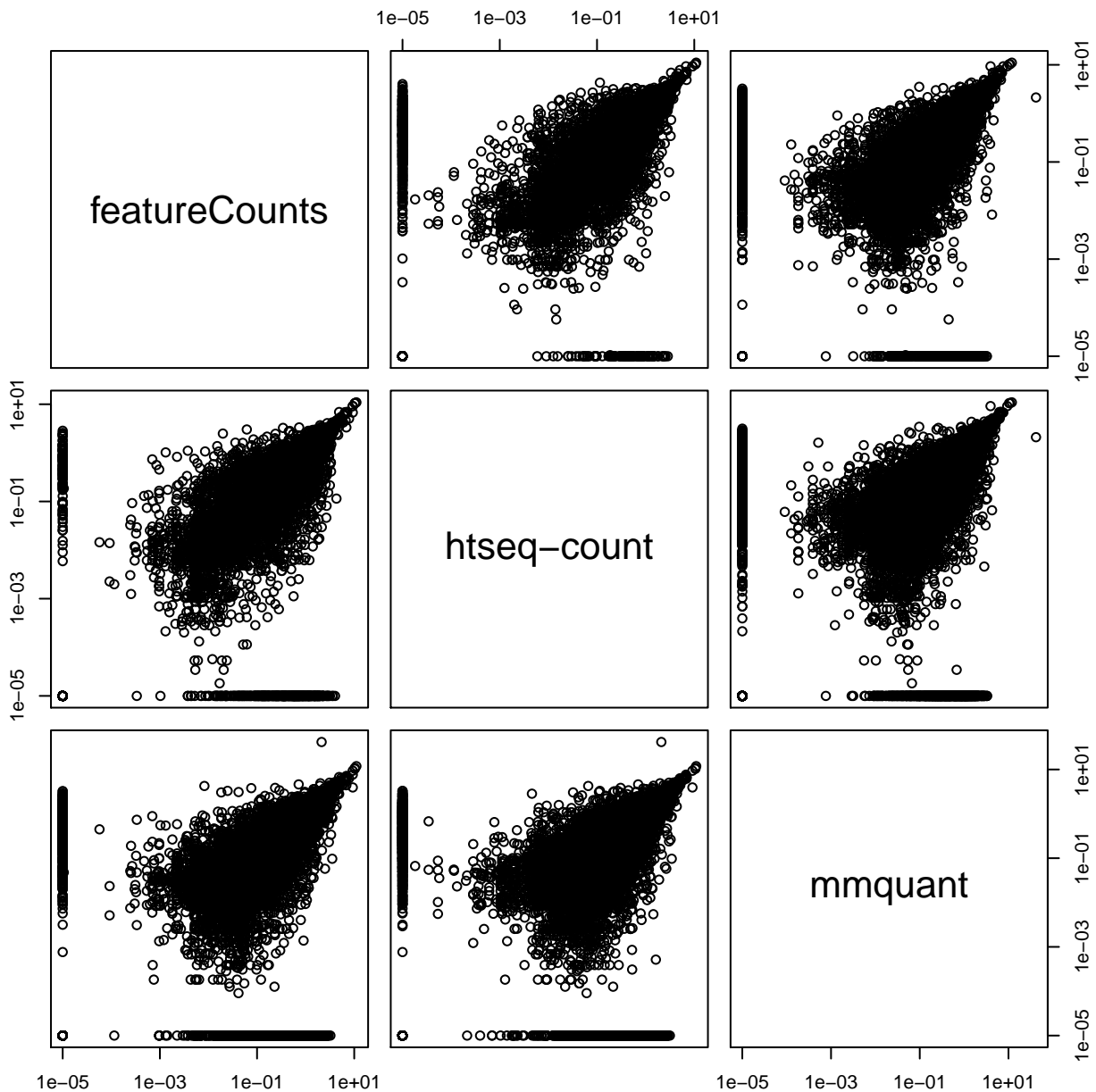

Supplement: Supplementary file 1 — Comparison of the p-value distributions. Each square outside of the diagonal compares a couple of tools. For instance, the top-right square compares featureCounts with mmquant. Each dot is a gene, its x-axis value is −log of the p-value given by a tool, whereas the y-axis value is −log of the p-value given by the other tool. In the aforementioned square, the value on the x-axis is given using the mmquant strategy, and the value on the y-axis is given using the featureCounts strategy. All axes are log-scaled, and p-values have been increased by 105 to render 0s. (PDF 1771.52 kb) [file 12859_2017_1816_MOESM1_ESM.pdf]
