# Supplementary material for: mmquant: how to count multi-mapping reads?
Source: BMC Bioinformatics. 2017 Sep 15;18:411. doi: 10.1186/s12859-017-1816-4 (PMC5603007; doi:10.1186/s12859-017-1816-4)
Supplement: Supplementary file 2 — List of differentially expressed merged genes, related to brain diseases. For each of the six “merged genes” potentially linked to brain diseases, we provide the actual genes they are made of, as well as their genomic loci. Notice that merged genes 3 to 6 involve two to three different loci. (PDF 48 kb) [file 12859_2017_1816_MOESM2_ESM.pdf]

Table 1 List of differentially expressed merged genes, related with brain diseases.

Merged gene	Gene Name	ENSEMBL Name	Locus
1	*ADK*	ENSG00000156110	10:74,151,185-74,709,303 (+)
	MRPL35P3	ENSG00000226253	10:74,527,584-74,527,947 (+)
	AC022540.1	ENSG00000232342	10:74,506,081-74,530,553 (−)
2	*HTRA2*	ENSG00000115317	2:74,529,377-74,533,348 (+)
	AUP1	ENSG00000115307	2:74,526,645-74,529,939 (−)
3	*GTF2I*	ENSG00000263001	7:74,657,667-74,760,692 (+)
	AC211433.1	ENSG00000232729	7:74,688,939-74,729,001 (−)
	GTF2IP4	ENSG00000233369	7:73,154,938-73,207,283 (+)
4	*hnRNP-A1*	ENSG00000135486	12:54,280,193-54,287,088 (+)
	AC021224.1	ENSG00000262477	18:32,412,182-32,413,236 (+)
	HNRNPA1P7	ENSG00000215492	18:32,412,214-32,413,176 (−)
	HNRNPA1P10	ENSG00000214223	19:11,666,069-11,667,030 (−)
5	*PKD1*	ENSG00000008710	16:2,088,710-2,135,898 (−)
	PKD1P5	ENSG00000254681	16:18,374,521-18,401,940 (−)
6	*RERE*	ENSG00000142599	1:8,352,397-8,817,643 (−)
	AC025884.1	ENSG00000258732	15:22,278,971-22,282,872 (+)
